# Editorial

**DOI:** 10.1120/jacmp.v2i4.2594

**Published:** 2001-09-01

This issue closes out Volume 2 of JACMP. I am pleased to note that during the year we introduced a couple of new features; Letters to the Editor and Book Reviews. Look for additional new features next year. We have had a 65% increase in the number of articles published over last year, and from the number of manuscripts currently under review we should see a similar increase next year.

In addition to the Editorial Board, whose hard work and dedication I greatly appreciate, this journal would not be possible without the many hours voluntarily given by the referees. I wish to acknowledge and thank them for their contributions. Over 100 individuals have acted as referees in the last two years and their names are listed alphabetically at the end of this editorial.

Important to the activities of the clinical medical physicist in the United States is the availability of certification by an appropriate governing body. After several years of careful thought and negotiation, two such bodies in the United States, the American Board of Radiology (ABR) and The American Board of Medical Physics, Inc. (ABMP), have reached a working agreement. This is of great importance to all clinical medical physicists in this country and I strongly recommend that they read the Letter to the Editor in this issue from the ABMP, which explains and contains this working agreement.

Once again I am very pleased to announce that the JACMP will continue as a subscription‐free journal for 2002 allowing it to have worldwide accessibility. This would not be possible without our commercial sponsors and advertisers. They are listed on the journal home page. Please take a moment to thank them and let them know that you appreciate being able to access the JACMP without charge.

Peter R. Almond, Ph.D.

Editor‐in‐Chief

